# Well-being in residency training: a survey examining resident physician satisfaction both within and outside of residency training and mental health in Alberta

**DOI:** 10.1186/1472-6920-5-21

**Published:** 2005-06-22

**Authors:** Jordan S Cohen, Scott Patten

**Affiliations:** 1 Department of Psychiatry, University of Calgary Faculty of Medicine Foothills Medical Center (Special Services Building) 1403-29^th ^St. NW Calgary, Alberta, Canada T2N 2T9; 2 Departments of Community Health Sciences and Psychiatry, University of Calgary. 1403-29^th ^St. NW Calgary, Alberta, Canada T2N 2T9

**Keywords:** Residency, physician, post-graduate well-being, stress, intimidation, harassment, and resources

## Abstract

**Background:**

Despite the critical importance of well-being during residency training, only a few Canadian studies have examined stress in residency and none have examined well-being resources. No recent studies have reported any significant concerns with respect to perceived stress levels in residency. We investigated the level of perceived stress, mental health and understanding and need for well-being resources among resident physicians in training programs in Alberta, Canada.

**Methods:**

A mail questionnaire was distributed to the entire resident membership of PARA during 2003 academic year. PARA represents each of the two medical schools in the province of Alberta.

**Results:**

In total 415 (51 %) residents participated in the study. Thirty-four percent of residents who responded to the survey reported their life as being stressful. Females reported stress more frequently than males (40% vs. 27%, p < 0.02). Time pressure was reported as the number one factor contributing to stress (44% of males and 57% of females). A considerable proportion of residents would change their specialty program (14%) and even more would not pursue medicine (22%) if given the opportunity to relive their career. Up to 55% of residents reported experiencing intimidation and harassment. Intimidation and harassment was strongly related to gender (12% of males and 38% of females). Many residents (17%) rated their mental health as fair or poor. This was more than double the amount reported in the Canadian Community Health Survey from the province (8%) or the country (7%).

Residents highly valued their colleagues (67%), program directors (60%) and external psychiatrist/psychologist (49%) as well-being resources. Over one third of residents wished to have a career counselor (39%) and financial counselor (38%).

**Conclusion:**

Many Albertan residents experience significant stressors and emotional and mental health problems. Some of which differ among genders. This study can serve as a basis for future resource application, research and advocacy for overall improvements to well-being during residency training.

## Background

Studies have shown medical school training to be a source of significant stress. The expectations and responsibilities only increase during residency training [1]. Although today's residents no longer live in their respective training hospitals, the pressures of residency are still extremely high. Residents are expected to be proficient clinicians, educators, researchers and administrators by the time they have completed their training. Improvements have been implemented in North America to identify, manage, and reduce resident stress [2]. Still, many residents report psychological symptoms during residency, feelings of becoming less humanistic and more cynical and "burning out" [3,4].

Researchers have attempted to examine and categorize stressors experienced by residents, which are experienced both within residency and in their personal life [5]. Still others have tried to predict levels of stress [6]. Resident responses to stress that have been described in the literature include: depression, burnout, anger/irritability, anxiety and substance abuse [5]. Resident stress has even been demonstrated at the biological level using cortisol measurements [7]. Others have reported changes in mood patterns from enthusiasm and depression, to anger and fatigue [8]. Sleep deprivation alone, has been shown to predispose residents towards more medical errors, injuries, increased alcohol and drug use, and increased conflict with other healthcare staff [9]. In the worst cases, resident suicides have occurred, forcing researchers to more closely examine residency stresses [10]. National wellness programs are attempting to improve resources for all physicians in Canada and changes are being made to individual residency programs [11,12]. In order to negotiate the stressors of residency, residents must become proficient "jugglers of the mind," maintaining the balance of all the complex biological, psychological and social interactions that occur during their training.

Differences have been reported in how males and females deal with stress [13,14]. Other groups have found no gender differences in stress response [15]. In some instances, the prevalence of symptoms that occur as reactions to stress is greater for residents than for the general population [16]. Many stress responses do not differ between these groups [8] However, residents are a unique group that are in many ways responsible for the health of the broader population and thus one might believe they should be more carefully scrutinized for such disorders as substance abuse. Involvement in stress management can lead to an overall more positive experience in medical residency [17].

The only Canadian study to date that has examined stress in residency training was performed by Toews and associates [12]. This study examined four of the medical schools in the country and did not identify any concerns with residents dealing with stress. To the best of our knowledge, our survey, known to PARA as "the Happy Doc study," is the first in Canada that will examine both stressors and well-being resources in residency. This paper presents findings from the "Happy Doc Study," referred to subsequently in this manuscript as "the study." The study was performed in each of two medical schools in Alberta during the 2003 academic year. It was our hope that by surveying members of the resident association we could increase our knowledge of current stressors effecting residents and determine what well-being resources are required, to improve residency training on a national level.

## Methods

### Survey composition

The study was conducted during the 2003 academic year among members of the Professional Association of Residents of Alberta (PARA). PARA represents all the resident physicians employed in the province of Alberta. The membership list from PARA thereby served as a sampling frame for this survey. The entire population indexed in the sampling frame was considered eligible for inclusion. This included residents in all levels of academic training. These residents were undergoing postgraduate training at both the University of Alberta and the University of Calgary. This was a voluntary study that PARA believed necessary to deal with the future well-being of its membership. The survey was distributed and collected via the PARA board members primarily during academic days, local mailboxes and e-mail. Surveys were distributed both by representatives within each specialty group and non-responders were followed up by e-mail versions of the survey. Unique identifiers were used in order to maintain the confidentiality of all participants. All of the data were collected over a six month period (January through June).

The survey required approximately twenty minutes to complete. The survey needed to be brief in order to maximize the response rate. Because the goals of the study were broad-based and descriptive, a decision was made to cover a large number of variables using single items and small modules, rather than including a restricted set of detailed "gold standard" measures. The questionnaire was divided into five sections: demographics, stress, intimidation and harassment, well-being and resources. Survey questions included qualitative rating scales, multiple responses and yes/no questions. To minimize a bias in rating scales responses (response acquiescence bias); the survey included a mixture of positively and negatively stated items [18]. Where numbers were large enough for valid statistical inference, groups were stratified by gender. The stress section contained questions regarding sources of stress as well as methods for dealing with stress (e.g. what would you say is the most important thing contributing to feelings of stress you may have?; thinking about the ways you deal with stress, how often do you do each of the following?). The term "stress" was not formally defined in the survey. The purpose of this was to measure "perceived stress" which might vary both quantitatively and qualitatively among individuals. For similar reasons we did not define the term intimidation and harassment as this is also perceived differently among individuals. We also decided to keep the two terms (intimidation and harassment) linked to avoid any confusion among resident when completing the survey. Percentages reported for intimidation and harassment among subgroups (e.g. inappropriate verbal comments) reflect overall percent of residents completing the entire survey and not those that reported intimidation and harassment, except where directly quoted in text (e.g. n = 65/170).

In certain instances group data was collapsed to increase numbers within response categories. Well-being questions (section D of results) were derived from the Canadian Community Health Survey (CCHS) so that results could be compared with members of the general Canadian population [19]. As a part of the study we have also included mental illness screening questions from the CCHS. Individuals that were positively screened for these mental disorders then underwent in depth questioning in the CCHS regarding diagnoses of these disorders. We have used these screening questions just to identify possible symptoms in residents. To avoid any stigmatization and thus decreased response rate, we did not further screen these individuals for psychiatric diagnoses.

Resources questions focused on knowledge of current resources (list provided of all resources and residents were asked which resource they were aware of prior to the survey), perceived need for future resources, and barriers and limitations towards residents seeking aid (e.g. If you were in a situation where you were experiencing an emotional or mental health problem, how would you deal with it?). In addition, questions were asked regarding career decisions (e.g. If possible, I would consider changing my residency program? [Yes/No]) and family physicians utilization.

### Statistics

Descriptive statistics were used to give an overview of the data as well as for comparison with results from a Canadian national survey, the Canadian Community Health Survey (CCHS) conducted by Statistics Canada . In cases where not all residents responded to individual questions, percentagesfall short of 100%. Confidence intervals, Chi-squares, and Fisher exact tests were used to compare differences between groups. p-values less then 0.05 were interpreted as indicating statistical difference. All percentages reported in this paper were rounded to the nearest whole number. In addition, decimal points were rounded to 2, except in cases where this would make the statistic difficult to comprehend (i.e. p-values < 0.001).

## Results

### (A) Demographics

The response rate for the survey was 51% (415/800). Of those residents who completed the survey, 47% (n = 195) were male and 52% (n = 217) were female. The median age was 29 years, with a range from 24–49 years. The marital status among residents revealed that 55% of residents surveyed were either married or in common-law relationships, 42% were never married, and 2% were widowed, separated or divorced. Among the residents in post-graduate programs, 40% graduated from an Alberta medical school, 45% graduated from a medical school within another Canadian province and 14% received their medical training from outside of Canada. Among all the levels of residency, 35% were from first year of post-graduate training, 30% were in second year, 15% were in third year, 8% were in fourth year, 10% were in fifth year, 2% were in sixth year and 0.2% were in their seventh year of training. The average number of hours worked per week among residents was 75 ± 16 hours. The number of hours worked ranged from 30 to 126 hours per week. There were no significant differences between genders with respect to marital status, residency specialty program, location of medical school training or mean number of hours worked per week.

### (B) Stress

The amount of stress in life on most days was determined using a five point scale (0 = not at all stressful, and 5 = extremely stressful). Many residents (34%) rated most days of their life as 4 to 5 on this scale. In the last 12 months of residency 43% of residents found this period of time to be "quite a bit" up to "extremely" stressful. Overall both genders reported a good degree of ability to handle unexpected and difficult stress. Fourteen percent reported their abilities as excellent, 56% good, 27% fair and only 3% of residents reported poor abilities to handle unexpected and difficult problems. Most residents reported an ability to handle day to day life demands (97% reported fair to excellent ability), with no differences between genders.

Residents were asked to rate the degree to which they agreed with how certain stresses relate directly to their residency program in the past 12 months. There were no significant differences between genders in these questions. Almost a third of residents (31%) either disagreed or strongly disagreed that their residency program allowed them freedom to decide how they did their job. Many of the residents (62% either agreed or strongly agreed) felt that their residency was very hectic. Residents disagreed or strongly disagreed (61%) that residency was free of conflicting demands that others made. More than one quarter of residents (27%) disagreed or strongly disagreed with having a lot to say about what happened in their residency. Over half of the residents (56%) either agreed or strongly agreed that there was pressure on examinations and evaluations. Two thirds of residents (66%) agreed or strongly agreed that there was insufficient sleep and frequent call. Many reported significant pressure due to their clinical workload (60% either agreed or strongly agreed). Many residents (17%) felt that there was stress due to high rates of death among patients (agreed or strongly agreed).

#### Contentment with career

When asked about their residency program, 14% of residence would consider changing their training program. In addition, more than one fifth (22%) of residents who participated in the survey reported that they would pursue another career if they had it to do all over again. When asked if they would change their career if they could live their lives over again, more males than females considered this change in program (18% vs. 11%, p < 0.02).

#### Gender differences

When categories were collapsed, females reported more significant stress than males (40% vs. 27%, p < 0.02). They also reported a higher degree of stress in the last 12 months (50 vs. 36%, p = 0.019). Overall males rated their abilities to handle unexpected and difficult problems better than females (p = 0.02).

When asked to think about stresses in day to day life, residents rated different conditions as a source of stress (see Table 1). Categories were collapsed to a 3 point scale (from original 7 point scale), from conditions causing less stress to more stress. Gender differences existed in time pressure, and caring for own children stress condition categories. Woman reported time pressure as being more stressful than males (79% vs. 64%, respectively, p < 0.001). However, more males reported caring for their own children as being either moderately or more stressful (11% and 8%, respectively) compared to females (4% and 9%, for moderate and more stress, respectively, p = 0.02).

**Table 1:** Rating of amount of stress in day to day life due to various conditions, separated by gender (actual numbers reported in brackets below percent responses).

**Possible sources of stress in day to day life**	**Less stress (%)**			**Moderate stress(%)**			**More stress (%)**		
	**Male**	**Female**	**Total**	**Male**	**Female**	**Total**	**Male**	**Female**	**Total**
Time pressure/not enough time ^a^	18% (36)	6% (14)	12% (50)	17% (33)	13% (29)	15% (62)	64% (124)	79% (171)	71% (295)
Own physical health problem or condition	77 (150)	78 (169)	77 (319)	11 (22)	8 (17)	10 (39)	11 (22)	14 (31)	13 (53)
Own emotional or mental health problem/condition	76 (148)	73 (160)	74 (308)	14 (28)	13 (29)	14 (57)	9 (17)	12 (25)	10 (42)
Financial situation	36 (70)	50 (108)	43 (178)	15 (29)	12 (26)	14 (55)	47 (92)	36 (78)	41 (170)
Own work situation	34 (67)	27 (59)	31 (126)	20 (39)	21 (46)	20 (85)	42 (83)	50 (109)	46 (192)
Residency program	40 (78)	43 (93)	41 (171)	24 (46)	25 (54)	24 (100)	35 (69)	31 (68)	33 (137)
Employment status	67 (131)	73 (158)	70 (289)	16 (31)	17 (36)	16 (67)	14 (28)	9 (20)	12 (48)
Caring for own children ^b^	38 (74)	40 (86)	39 (160)	11 (22)	4 (8)	7 (30)	8 (16)	9 (20)	9 (36)
Caring for others	66 (129)	67 (145)	66 (274)	19 (37)	11 (24)	15 (61)	8 (15)	8 (18)	8 (33)
Other personal or family responsibilities	56 (109)	57 (124)	56 (233)	18 (36)	17 (37)	18 (73)	24 (46)	24 (51)	24 (97)
Personal relationships	53 (103)	56 (122)	54 (225)	25 (48)	21 (45)	22 (93)	21 (40)	23 (49)	22 (89)
Discrimination	81 (157)	83 (181)	82 (338)	8 (16)	9 (19)	8 (35)	8 (15)	6 (13)	7 (28)
Personal and family safety	84 (163)	88 (192)	86 (355)	8 (16)	6 (14)	7 (30)	5 (9)	5 (10)	5 (19)

^a ^Females more stressed by time pressure (p < 0.001)

^b ^Males more stressed by caring for own children (p < 0.020)

In addition, respondents had the opportunity to report any other conditions that contributed to their daily stress. These conditions included: the travel time required in rural medicine, large amount of information to learn, hospital politics between services, living separated from spouse, marital relationship, motivation, responsibilities at work, examinations, balancing learning and clinical service components of residency and illness in family members. When asked to rank the most important thing contributing to feelings of stress, both males and females ranked time pressure as their number one choice (see Table 3). Other highly ranked contributors to stress included financial situation, own work situation, residency program, residency program and personal relationships.

**Table 3:** Top resident ranked contributors to feelings of stress during residency between genders.

**Contributor to stress**	**Males**		**Females**		**Totals**	
	**Percent (%)**	**Rank**	**Percent (%)**	**Rank**	**Percent (%)**	**Rank**
Time pressure	44%	1	57%	1	51%	1
Financial situation	25	2	11	3	18	2
Own work situation	11	3	12	2	12	3
Residency program	7	4	9	4	8	4
Personal relationship	7	5	6	5	6	5

#### Dealing with stress

The questionnaire included a battery of questions about ways residents deal with stress. This was rated on a 4 point scale from 4 = never to 1 = often, which was collapsed to a 3 point scale for analysis (see Table 2). There were many residents that used positive approaches in dealing with stress (e.g. by talking to others). However, a significant amount of residents dealt with stress in less productive ways (e.g. by avoiding others).

**Table 2:** Frequency of the ways residents reported dealing with stress, separated by gender (actual numbers reported in brackets below percent responses).

**Frequency of occurrence (%):**	**Often**			**Sometim es**			**Rarely/ never**		
	**Male**	**Female**	**Total**	**Male**	**Female**	**Total**	**Male**	**Female**	**Total**
Talk to others	48^%a ^ (94)	80% (172)	64% (266)	44%^b ^ (84)	18% (38)	29% (122)	8% (15)	3% (5)	5% (20)
Avoid being with people	10 (19)	5 (11)	7 (30)	46 (89)	48 (105)	47 (194)	44 (85)	46 (100)	44 (185)
Sleep more than usual	9 (18)	12 (25)	10 (43)	33 (64)	37 (81)	35 (145)	57 (112)	51 (111)	54 (223)
Try to feel better by eating more or less ^c^	5 (10)	18 (39)	12 (49)	31 (60)	39 (83)	35 (143)	64 (123)	43 (92)	52 (215)
Try to feel better by smoking more	2 (3)	0.46 (1)	1 (4)	2 (5)	1 (2)	2 (7)	25 (49)	21 (46)	23 (95)
Try to feel better by drinking alcohol ^d^	3 (5)	0.46 (1)	1 (6)	18 (35)	12 (26)	15 (61)	79 (154)	87 (188)	83 (342)
Try to feel better by using drugs/meds ^e^	0.49 (2)				3 (14)				95 (391)
Try to look on the bright side of things	44 (85)	53 (115)	48 (200)	47 (91)	38 (81)	42 (172)	8 (16)	9 (19)	8 (35)
Exercise	47 (91)	43 (94)	45 (185)	33 (65)	36 (78)	35 (143)	19 (37)	21 (45)	20 (82)
Pray or seek spiritual help	20 (39)	24(51)	22 (90)	28 54)	21 (46)	24 (100)	52 (101)	55 (119)	53 (220)
Relax by doing something enjoyable	47 (92)	49 (106)	48 (198)	43 (84)	42 (91)	42 (175)	9 (18)	8 (17)	8 (35)
Blame yourself	8 (15)	12 (25)	10 (40)	32 ^f ^ (62)	52 (113)	42 (175)	60 (96)	36 (77)	46 (173)
Wish the situation would go away	19 (37)	24 (52)	22 (89)	45 (87)	56 (121)	50 (208)	35 ^g ^ (69)	20 (43)	27 (112)

^a ^Males talk to often others less than females (95% CI, 0.41–0.56 vs. 0.74–0.85).

^b ^Males talk sometimes to others more than females (95% CI, 0.37–0.51 vs. 0.13–0.23).

^c ^Females more often change eating habits compared to males (p < 0.001).

^d ^There was a significant difference between those who drink often/sometimes and those who drink rarely/never (95% CI, 0.13–0.20 vs. 0.80–0.87).

^e ^Numbers were too small to stratify by gender.

^f ^Males blame themselves sometimes less than females (95% CI, 0.26–0.39 vs. 0.46–0.59).

^g ^More males rarely wish the situation will go away compared to females (95% CI, 0.21–0.34 vs. 0.11–0.21).

### (C) Intimidation and harassment

**Figure 1:**
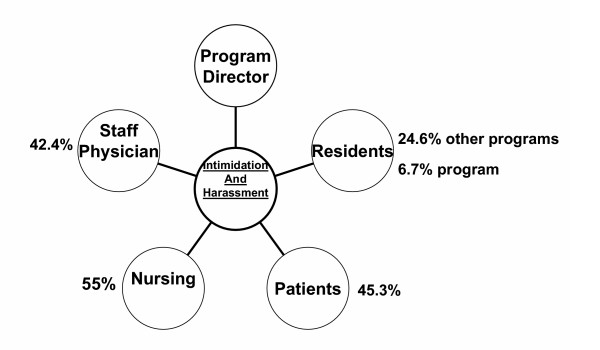
*Sources of intimidation and harassment as reported by residents in form of percent response. *Note: respondents had the opportunity for multiple responses, accounting for the total percent response exceeding 100%.

More than two-thirds of residents responding to the survey (n = 302; 73%) reported experiencing intimidation and harassment. Residents reported intimidation and harassment from many members of the healthcare team. The highest degree of reported intimidation and harassment was experienced from nurses (n = 166; 55%) (Fig. 1). In addition to our suggested categories, residents also had the opportunity to mention other sources of intimidation and harassment. Forty percent of residents reported intimidation and harassment from families of patients (n = 121). Many residents (n = 42; 14%) reported intimidation and harassment from secretaries/unit clerks.

**Figure 2:**
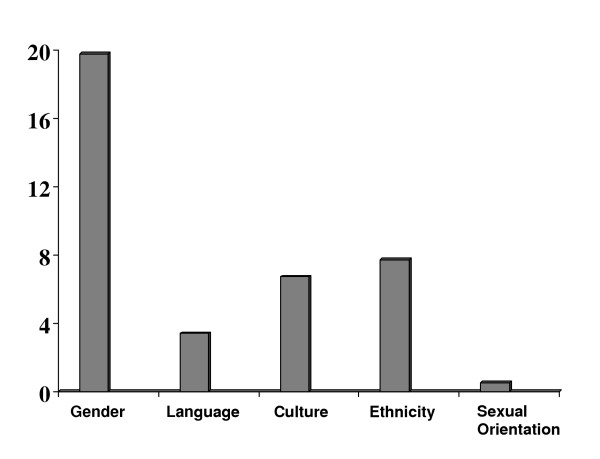
*Percentage distribution of the perceived bases for resident intimidation and harassment.* The Y-axis has been collapsed for better distinguishing differences among groups, but it should be noted that this only reflects 20% of the residents responding to the survey. As in Figure 1, multiple responses were possible from each resident.

Over two thirds (n = 273; 66%) of residents reported intimidation and harassment in the form of inappropriate verbal comments. Inappropriate or unwanted physical contact was experienced by (n = 21; 5%) of residents. Sexual harassment was reported by (n = 18; 4%) of residents. Work given as punishment was reported as a form of intimidation and harassment by (n = 39; 9%) of residents. Other residents reported that privileges/opportunities were taken away as their form of intimidation and harassment (n = 26; 6%). Finally, 3% (n = 10) of those reporting intimidation and harassment reported it occurring in the form of recrimination for reporting these incidences. Residents were questioned on what was the basis for the reported intimidation and harassment (Figure 2). Similar to Figure 1, multiple responses were permissible. The primary reported basis for intimidation and harassment was gender (n = 17/123, 12% of males; and n = 65/105, 38% of females). Due to the smaller sample sizes we were unable to look for statistical differences between genders in responses to these questions. Of the residents that reported intimidation and harassment 51% (n = 211) stated that this had occurred more than once. Only 52% (n = 215) of residents who reported intimidation and harassment were aware of the process to address this issue and only 44% (n = 181) of residents felt this process was adequate, fair and independent.

### (D) Well-being

The results for life satisfaction and self-rated mental health used in this survey were compared to the CCHS estimates for the province's (Alberta) population in Tables 4 and 5. CCHS estimates are for people in the 25 to 64 year age group. Most residents (78%; 74% of males, 82% of females) reported their satisfaction with life in general as either satisfactory of very satisfactory. This was lower than CCHS results for both the province of Alberta (85%) and for the entire country (85%). Seven percent of residents reported being dissatisfied or very dissatisfied with their life in general (7% of males, 6% of females). This was slightly higher for men than the CCHS results for Alberta (6% for males and 6% of females) and the country (5% of males and 5% of females).

**Table 4:** Comparison of relative frequencies of life satisfaction results between residents from the study and the provincial population from the CCHS.

**Canada (%)**	**Very Satisfied**		**Satisfied**		**Neither satisfied nor dissatisfied**		**Dissatisfied or very dissatisfied**	
*Alberta*	*TS*	*CCHS*	*TS*	*CCHS*	*TS*	*CCHS*	*TS*	*CCHS*

Males	24%	29%	50%	55%	18%	10%	7%	6%
Females	21	35	61	52	12	8	6	6
Totals	22	32	56	54	15	9	6	6

CCHS = Canadian Community Health Survey, TS = The Study

**Table 5:** Comparison of relative frequencies of self-rated mental health results between residents from the study and the provincial population from the CCHS.

**Canada (%)**	**Excellent**		**Very good**		**Good**		**Fair or poor**	
*Alberta*	*TS*	*CCHS*	*TS*	*CCHS*	*TS*	*CCHS*	*TS*	*CCHS*

Males	21%	27%	30%	35%	32%	31%	16%	7%
Females	14	23	30	36	38	32	18	9
Totals	17	25	30	35	35	32	17	8

CCHS = Canadian Community Health Survey, TS = The Study

Overall residents rated their mental health lower than CCHS data for both Alberta and the country. More male residents rated their mental health as excellent (20%) than females (14%). A similar discrepancy between genders occurred in the CCHS (26% of males and 23% of females in Alberta; and 30% of males and 26% of females throughout the country). Many more residents reported their mental health as fair or poor (17%) compared to CCHS data from the province (8% for Alberta) or the country (7%).

Table 6 show the percentage of residents that would require further screening for mental disorders based upon the results of symptoms screening questions from the CCHS for specific mental disorders. It should be noted that being positively screened does not infer a resident will have the disorder. The proportions screening positive was much smaller than the national CCHS data.

**Table 6:** Percentage of Residents that would require further screening for mental disorders based upon the results of symptoms screening questions from the CCHS* for specific mental disorders.

**Type of mental disorder**	**% Males screening positive for disorder**		**% Females screening positive for disorder**	
	**"The Study"**	CCHS 1.2*	**"The Study"**	**CCHS 1.2**

Depression	30%	49%	35%	55%
Panic disorder	9	40	16	49
Bipolar Disorder	7	20	12	19
Generalized anxiety	5	40	12	48
Social Phobia	12	20	15	22
Agoraphobia	2	13	3	20

*CCHS = Canadian Community Health Survey. This project was a component of the World Mental Health 2000 project. The estimates are based on a national sample 15 years of age or over, weighted to national household resident demographics (n = 36,984), for more information, see .

Over half of residents reported having a family physician (55%). More female residents have a family doctor compared to their male counterparts (68% vs. 41%, p < 0.001). Of those who reported having a family doctor, 68% have had an appointment in the last 12 months. Again, more female residents (76%) than males (54%) reported visiting their family physician (p < 0.001).

### (E) Resources

There was a large variation in resident awareness of the well-being resources available to them in Alberta (see Table 7). The majority of residents (86%) identified their program director as a possible resource. This was the only resident resource that over 80% of residents were aware of. Over one third of residents wished to have a career counselor (39%) and financial counselor (38%). Approximately one fourth of residents would like a program ombudsman (26%) and resident support group (24%). Residents were asked to rate well-being resources on a seven point, scale (1 = not important, 7 = extremely important). Based on this scale, resident colleagues (67%), program directors (60%) and external psychiatrist/psychologist (49%) were rated as extremely important most frequently. When asked to rank the top three well-being resources a resident would use if they were in a situation where they were experiencing an emotional or mental health problem, resident colleagues were ranked as the number one choice, followed by program director and external psychiatrists and psychologists.

**Table 7:** Resident awareness of well-being resources within the province of Alberta.

**Resource**	**Number**	**Percent (%)**
Telephone hotline	258	63%
Physician and family support group	294	71
Resident advocate	264	64
External psychiatrist or psychologist	156	38
Emergency consultation service	118	29
Program director *	354	86
Chief resident	218	53
Resident colleague	280	68
Health region	59	14
University	59	14

* Identified as most recognized well-being resource by residents completing the survey.

Unfortunately, 9% stated they would not seek help for emotional or mental health concerns. Reasons for not seeking help included (summarized into categories): the ability and desire to handle these situations on their own or with assistance exclusively from family or friends, fear of repercussion or recrimination, stigma/embarrassment/social stigma, accessibility (time, money), and issues of confidentiality. Estimates from the CCHS for those who responded that they had not sought help for an emotional or mental health issue in the past 12 months because of acceptability issues (i.e. competing demands on time, attitudes towards illness, health care providers or the health care system) indicated that 4% of were in this category.

Residents were asked to choose one or more ways that they would deal with a fellow resident who was experiencing an emotional health problem. The majority of residents would suggest that the resident get help (85%). Many residents would offer to go with the resident for help (76%). A quarter of residents would contact their program director (25%). Less than one in ten residents would notify the Post-Graduate Medical Education office (2%), the provincial resident association (8%), the Royal College of Physicians and Surgeons (1%), and the Alberta Medical Association (3%). Four percent of residents responded that they would do nothing.

## Discussion

While the rate of return (51%) was disappointing, it is comparable to other studies of this type [20]. More than two-thirds of responders (67%) to the survey were in first or second year of training. Thus, the results are more representative of residents who are less experienced in their training. Less experience might lead to increased stress. This possible response bias may have lead to an increased reporting of stress. However, residents that are more senior in training have other stresses that may be equally concerning (e.g. final examinations, higher expectations). The number of hours worked per week (75 ±16) was consistent with previous surveys of the PARA membership. In addition, since the survey was performed over a period of six months (January to June, 2004) it is possible that responses were biased based on the higher prevalence of stressors, such as depressed mood during the winter months.

Compared to previous Canadian studies of resident stress in which self-reported stress was not stated as a concern [14], residents in the study reported most days of their life as stressful (34%). Similar to the research done by Toews and associates [14], we also found that females reported a higher degree of stress than males (40% vs. 27%, p < 0.02). This may be due to stresses that are unique to the female gender [21]. One must also wonder whether the results are due to a reporting bias, in that females tend to be more open about their stress than their male counterparts.

Unfortunately, residents also had difficulties dealing with stress and resorted to more troublesome behaviors. A significant amount of residents reported often turning to alcohol to deal with stress and just less than 5% reported using drugs or medication to feel better (sometimes or often). These numbers are not significantly different than the population, but are quite concerning when considering the responsibilities of this group of professionals [8].

A large portion of the residents surveyed would consider changing their programs if given the opportunity. This speaks to the need for post-graduate medical education to ensure there is increased flexibility in residency, by taking measures such as increasing the amount of re-entry positions. Even more concerning was that over one fifth of residents reported that they would pursue another career if they had it to do all over again. Clearly, this speaks to the need to improving resident well-being in training.

Many residents reported experiencing intimidation and harassment. This result is consistent with the studies examining resident bullying [20]. The main form of this intimidation and harassment was in inappropriate verbal comments (66%). These results seem quite different from a previous study of psychiatry residents in Edmonton, Alberta, which concluded that intimidation in the psychiatric educational environment was not a significant issue [23]. However, due to the setup of our study we did not choose to stratify results from individual programs and therefore cannot directly comment on the psychiatry residents results. The primary basis reported for the intimidation and harassment was gender (12% of males and 38% of females), a difference that did not attain statistical significance, possibly because of the small sample size. However, it is quite possible that intimidation and harassment is one of the reasons that females in our survey reported more stress. This difference is consistent with recent publications that revealed increased female reporting of resident bullying [22,24]. Intimidation and harassment occurred often multiple times (more than once in 52% of those responding to the study) in both genders. Over half of the residents felt that the process to deal with it was not adequate, fair and independent. This speaks to the need for further educating all individuals in the healthcare system on resident well-being.

It appears that although the majority of residents are quite resilient to all of life's stresses during training. However, there is a significant group that seems to be having difficulty with their own well-being during this period of their lives (i.e. fair or poor life satisfaction and rated mental health), possibly to an extent below the levels in the general population.

Due to our study's design, we cannot predict what proportion of individuals had a mental illness, nor compare rates to the normal population in the province or country. However, ratings obtained with the CCHS screening questions did not suggest a higher prevalence of disorders. It would not be surprising if the prevalence of specific disorders were lower in this highly selected professional group than in the general population. The results suggest, however, that non-illness-related issues represent the main difference between residents and the general population. Another well-being concern was that many residents still do not have a family physician and a significant amount did not use them (no appointment in the twelve months prior to being surveyed). With all the stresses or residency and the potential for decreased physical health and well-being, the need for more residents to acquire a family physician to be available for dealing with such issues is crucial [25]. The Canadian Psychiatric association has position statements both on the treatment of mentally ill physicians and on trainee safety [25,26]. Some of the recommendations include: 1) that any physician with a possible psychiatric illness should receive an assessment quickly, ideally by a psychiatrist who is not a colleague or friend; 2) that the treating psychiatrist must urge the physician-patient to obtain a family physician as soon as possible and aid in this process as necessary; and 3) all provinces should have psychiatrists who serve on, or consult to, their physician well-being committees. In addition, the trainee safety position statement recommends that minimum standards exist for resident safety and that there should not be any coercion of trainees to see potentially violent patients.

Based on the wide variation of awareness of many well-being resources more education should be applied to this area. Resident career and financial counseling were the highest ranked well-being resource. This was likely rooted in the fact, that many residents's reported high reported stress due to financial situation and the dissatisfaction with residency training and the medical profession. Resident colleagues, program directors and psychiatrist/psychologist(s), were the top resources residents preferred in times of emotional or mental health need. There is a definite need to properly train and educate program directors and all residents in how to deal with well-being concerns. Adequate psychiatric/psychological aid to residents in the province must be an important priority [25]. The majority of residents reported that they would intervene to aid a colleague having emotional difficulties. Most often by suggesting they go for help (85%) or by offering to go with them for help (76%). Only a small portion of the residents would inform any medical organization. This may suggest that while residents want to help their peers, they prefer to do so in ways that do don't involve notifying external guarding bodies.

## Conclusion

It is clear that there are significant stressors incurred during residency. Some of which have differences between genders. Intimidation and harassment occurs among many residents. It is also important to recognize that a significant amount of residents are vulnerable to emotional and mental health concerns. Residents need to be better informed about well-being resources. It is not clear as to whether the resources resident's perceived to be important are the necessary aids to deal with well-being concerns during training. However, ensuring the education of other healthcare professionals in the area of well-being is needed, so that resident's who ask for help will be directed the correct sources.

Now that this pilot study is complete we are working on administering the happy doc survey throughout the entire country. Once this data is collected comparisons can be made inter-provincially. Results of this collaboration can be used to help identify key stressors in residency and develop well-being resources for the future.

## Abbreviations

AMA: Alberta Medical Association

CCHS: Canadian Community Health Survey

PARA: The Professional Association of Residents of Alberta

TS: The Study (referring to the survey presented in this paper).

## Authors' contributions

JC conceived the study, and participated in its design and coordination and drafted the manuscript. He is a physician in residency training in Psychiatry at the University of Calgary. He is the past president of PARA and past member on the executive of the Canadian Association of Interns and Residents (CAIR).

SP participated in the design of the study, read and approved all stages of the manuscript.

## Pre-publication history

The pre-publication history for this paper can be accessed here:



## Supplementary Material

Additional File 1 The complete version of the resident physician questionnaire utilized for this study - can be referenced in text in the methodology as soon as any ref. made to survey/questionnaire. Click here for file
